# Dietary Bovine Lactoferrin Reduces the Deleterious Effects of Lipopolysaccharide Injection on Mice Intestine

**DOI:** 10.3390/nu16234040

**Published:** 2024-11-26

**Authors:** Anne Blais, Natsuko Takakura, Marta Grauso, Caroline Puel-Artero, François Blachier, Annaïg Lan

**Affiliations:** 1 UMR-PNCA, Université Paris-Saclay, AgroParisTech, INRAE, 91120 Palaiseau, France; natsuko.mikogami@gmail.com (N.T.); marta.grausoculetto@cea.fr (M.G.); francoismichel.blachier@gmail.com (F.B.); annaig.lan@agroparistech.fr (A.L.); 2 Soredab, LDT, La Tremblaye, 78125 La Boissière-Ecole, France; caroline.puel@sorebad.org

**Keywords:** lactoferrin, lipopolysaccharides, intestinal inflammation, intestinal dysfunction

## Abstract

Background/Objectives: Injection of lipopolysaccharides (LPS) in experimental models induces a systemic inflammatory response that is associated with deleterious effects on intestinal morphology and physiology. In this study, we have studied in female mice the effects of dietary supplementation with bovine lactoferrin (bLF) given before intraperitoneal injection of LPS on jejunum and colon. Methods: The first study evaluated the efficiency of different bLF and LPS concentrations to determine the optimal experimental conditions. For the second study mice were fed with 1% bLF before the LPS challenge (3 mg/kg body weight). Plasmatic markers of inflammation, intestinal morphology, permeability, and expression of genes related to epithelial differentiation, epithelial barrier function and intestinal inflammation in both small intestine and colon were evaluated. Results: bLF ingestion before the LPS challenge reduced the TNF-α circulating concentration, compared to control animals. This decrease in plasma TNF-α was correlated with improved intestinal permeability. The morphology of jejunal epithelium, which was affected by LPS challenge, was partly maintained by bLF. Measurement of the expression of genes encoding proteins involved in epithelial differentiation, intestinal inflammation, and epithelial barrier function suggests an overall protective effect of bLF against the adverse effects of LPS in the jejunum. In the colon, the effects of bLF ingestion on the subsequent LPS challenge, although protective, remain different when compared with those observed on jejunum. Conclusions: Taken together, our data indicate that bLF dietary supplementation does have a protective effect on the deleterious intestinal alterations induced by LPS systemic inflammation.

## 1. Introduction

Lactoferrin (LF) is an 80 kD sialylated iron-binding glycoprotein of the transferrin family which is found at high concentrations in human breast milk [1]. LF is also present in various mucosal secretions and, to a lesser extent, in blood, originating from secretion by neutrophils. LF is acting in the host non-specific defense against invading pathogens [2]. As reviewed by multiple groups, LF has numerous biological functions, including antimicrobial, anti-inflammatory, immunomodulatory and anti-oxidative activities [3,4]. When orally administered, LF has been also shown to have several protective activities in animals and humans [5,6]. In the gastrointestinal tract, orally administered LF exerts a stimulating effect on the growth and differentiation of intestinal epithelial cells in vivo [7,8,9,10]. LF also modifies cytokine and antibody productions by intestinal immune cells in healthy mice [11,12]. In addition, several studies have shown that LF administration can reduce the severity of intestinal inflammation. Indeed, in animal colitis models, LF oral administration alleviated symptoms of inflammatory bowel disease by reducing pro-inflammatory cytokines in the colonic tissue [13,14]. Recently, Hu et al. reported that oral LF administration attenuates intestinal barrier dysfunction and inflammation induced by ingestion of a mycotoxin [15].

Furthermore, the effectiveness of LF to alleviate lipopolysaccharides (LPS) induced inflammation has been reported in different in vivo conditions [16,17,18,19]. This anti-inflammatory effect appears mediated by the ability of LF to bind and sequester LPS [20]. LPS is a component of the wall of Gram-negative bacteria that initiates an acute systemic inflammatory response including hypotension found in early stages of septic shock [21].

The in vivo administration of LPS is a well-established model of endotoxemia, because its injection induces a systemic inflammatory response including finally widespread organ dysfunctions [22]. At the intestinal level, LPS systemic administration increases intestinal permeability, epithelial cell apoptosis and shedding, intestinal villus shortening and diarrhea [23,24,25,26] and decreases intestinal mucosa oxygen consumption [27] as well as intestinal amino acid absorption [28].

In this context, the aim of the present study was to determine whether LF intake has beneficial effects against systemic and local inflammation. To this end, we evaluated the efficiency of bovine LF (bLF) dietary supplementation given for 7 days before LPS challenge to alleviate systemic inflammation and to prevent the intestinal deleterious effects of inflammation in female Balb/c mice. Plasmatic markers of inflammation, intestinal morphology, permeability, and expression of genes related to epithelial differentiation, epithelial barrier function and intestinal inflammation in both small intestine and colon were evaluated 6 h and 24 h after the LPS challenge.

## 2. Materials and Methods

### 2.1. Experimental Design and Diets

Nine-week-old female Balb/c strain mice (Envigo, Gannat, France) were acclimated for 1 week with free access to a standard AIM-93 modified P14 casein diet (Table 1) and tap water. Each mouse was maintained in a cage under controlled conditions of temperature (23 °C), humidity (55 ± 10%), and light (12:12-h light-dark cycle). Animal experiments were conducted according to the European legislation on animal experimentation, validated and approved by the Ethics Committee in Animal Experiment INRAE Jouy-en-Josas and French Research Minister (APAFiS #10765-2017060217335487).

LPS from *Escherichia coli* O111:B4 purified by ion-exchange chromatography (Sigma-Aldrich, St-Quentin-Fallavier, France) was diluted in sterile saline. Bovine milk-derived LF (>95% protein purity, approximately 10% iron saturated; Armor Protéines, St-Brice en Coglès, France) and/or acid casein were incorporated to the above mentioned P14 diet to obtain 0, 2.5 and 10 g of bLF per kg of diet (CT, LF 0.25% and LF 1% respectively). The diet compositions are shown in Table 1. After LPS or saline injection, steel wire net floor was used to observe and collect the stool.

**Figure 1:**
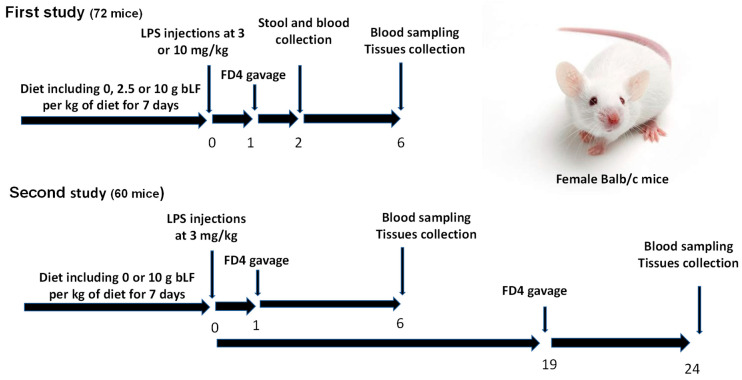
Schematic representation of the experimental design. The first study included 3 groups of 24 mice fed with LF 0, LF 0.25% or LF 1% diet for 7 days. At t0, 8 mice of each group were intraperitoneally (i.p.) injected with sterile phosphate buffered saline (PBS) or lipopolysaccharide (LPS) solution at a dose of 3 or 10 mg/kg of bw. The second study included 2 groups of 30 mice fed with LF 0 or LF 1% diet for 7 days. At t0, 10 mice of each group were i.p. injected with PBS and 20 mice with a LPS solution at 3 mg/kg of bw. LPS-treated mice were euthanized either 6 h or 24 h after LPS injection. The untreated mice receiving saline injection were euthanized after 24 h.

Figure 1 shows the experimental protocol used in this study. The first study evaluated the efficiency of different bLF and LPS concentrations to determine the optimal experimental conditions. Mice were randomly divided into three groups of 24 individuals and fed without LF (LF 0), or with LF 0.25% or LF 1% diet for 7 days. On day 8, 8 mice of each group were intraperitoneally (i.p.) injected with sterile phosphate buffered saline (PBS) or LPS diluted in PBS at a dose of 3 or 10 mg/kg body weight (bw). One hour later, each mouse received Fluorescein-isothiocyanate-conjugated dextran 4000 (FD4, Sigma-Adrich, St. Louis, MO, USA) at 500 mg/kg bw by oral gavage. Two hours after the LPS or saline injection, blood samples were obtained by retro-orbital bleeding. Six hours after LPS or saline injection, blood was withdrawn by intracardiac puncture under anesthesia by inhalation of isoflurane and tissues were collected. Blood was collected in EDTA tubes, and plasma was frozen and stored at −80 °C.

The second experiment included 60 mice randomly divided into two groups of 30 individuals fed with the diet including or not 10 g/kg of bLF for 7 days, respectively. On day 8, 10 mice of each group were i.p. injected with PBS and the other 20 mice of each group were i.p. injected with a LPS solution at a dose of 3 mg/kg bw. LPS-treated mice were euthanized either 6 h or 24 h after LPS injection. The untreated mice receiving saline injection were euthanized after 24 h. Five h before euthanasia, each mouse received FD4. Blood was withdrawn by intracardiac puncture under anesthesia by inhalation of isoflurane and intestinal tissues (jejunum and colon) were collected. Blood was collected in EDTA tubes, and plasma was frozen and stored at −80 °C.

### 2.2. Stool Water Content

To evaluate the severity of diarrhea, the feces water content was measured according to the following procedure. Two h after LPS or saline injection, the feces were collected in the 2.0 mL microtube directly, weighed and stored at −80 °C with a sealing film. Feces were dried using a vacuum dryer and weighed again after total drying, to calculate the water content.

### 2.3. In Vivo and Ex Vivo Gut Permeability Assessment

Fluorescein-isothiocyanate (FITC)-conjugated dextran 4000 (FD4) was diluted to 50 mg/mL in saline and was administered at 10 mL/kg bw (500 mg/kg bw) by gavage 5 h before blood collection to evaluate in vivo permeability. Plasma fluorescence (FITC) was measured from blood collected at 6 h or 24 h after LPS/saline injection.

To assess potential changes in the intestinal barrier function, ex vivo electrophysiological and epithelial permeability measurements were performed in Ussing chambers (EasyMount, Physiologic Instrument Inc., San Diego, CA, USA) as previously described [29] with the following modifications. Mice proximal colon and jejunum samples were opened along the mesenteric line and mounted on an insert with an exposed area of 0.1 cm^2^. Mounted tissues were clamped at 0-mV to record the short-circuit current (I_sc_, uA/cm^2^) and left to equilibrate 30 min before trans-epithelial electrical resistance (R_t_, ohm*cm^2^) measurement. To evaluate paracellular permeability, FD4 (Sigma-Aldrich) was added to the mucosal side at the final concentration of 0.25 mg/mL. After 90 min, FITC fluorescence flowed in the serosal side chamber was measured with a spectrophotometer. Tissue viability at the end of each experiment was verified with carbachol (CCh, 10^−4^ M), applied at the serosal side of the tissue, looking for the activation of the calcium-dependent chloride secretion by the I_sc_ increase.

### 2.4. Quantification of Gene Expression via Real-Time Polymerase Chain Reaction (qRT-PCR)

The samples were kept at −80 °C, until total RNA extraction using TRIzol reagent, after homogenization with a Tissue lyser (Qiagen SAS, Courtaboeuf, France). RNA concentrations in samples were measured with a NanoDrop ND-1000 UV-Vis spectrophotometer. RNA was purified using an RNeasy Minikit (Qiagen) and DNase I treatment. Total RNA was reverse transcribed using a high-capacity cDNA kit protocol (Life Technologies, Courtaboeuf, France). qRT-PCR was performed with Fast SYBR Green MasterMix (Thermo Fischer Scientific, Courtaboeuf, France), using gene specific primers (sequences available on demand) and the StepOne Real-Time PCR system (Applied Biosystems, Life Technologies, Villebon-sur-Yvette, France) as previously described [30]. Gene expression was determined using the 2^−∆∆Ct^ formula, where ∆∆Ct = (Ct target gene—Ct reference gene) using *Hprt* as the house-keeping gene.

### 2.5. Histological Analysis

Segments of the jejunum were fixed in 4% buffered formaldehyde for histological analysis. Jejunum sections stained with hematoxylin, eosin and safran were coded for blind microscopic assessment. Length of well-oriented villus was determined by image analysis using NDP.view2Plus software (U12388) (Hamamatsu Photonics Ltd., Iwata City, Japan).

### 2.6. Protein Quantification by ELISA

Bovine lactoferrin concentration was measured in plasma using a Bovine Lactoferrin ELISA Kit (Bethyl Laboratories, Montgomery, TX, USA). Plasma TNF-α was determined using a sandwich ELISA kit (Thermo Fischer Scientific, Courtaboeuf, France) according to the manufacturer’s instructions. (U12388).

### 2.7. Statistical Analyses

All data are expressed as means ± SEM and compared using a two-way or a one-way analysis of variance (ANOVA) and a Tukey multiple comparison test. A *t*-test was also used to assess differences between two treatments at each time point. Significance was established at *p* < 0.05. Correlations were analyzed by Pearson’s correlation test. All statistical analyses were performed using Prism^®^ Version 6.05 (GraphPad Software Inc., San Diego, CA, USA).

## 3. Results

### 3.1. Selection of bLF and LPS Doses Based on Plasmatic TNF-α Concentration, Intestinal Permeability and Diarrhea Severity

The first experiment evaluated the effects of bLF supplementation for 7 days at two different doses (LF 0.25% or LF 1% diet, respectively 2.5 and 10 g of bLF per kg of diet) when LPS was i.p. injected at 3 or 10 mg/kg bw. The day before the LPS injection, we evaluated the bLF plasma concentration 1 h after ingestion of a 1 g meal including bLF. Plasmatic bLF values of 109 ± 10 and 505 ± 51 ng/mL were obtained when mice ingested respectively a meal including 0.25% or 1% bLF indicating that the plasmatic concentration of bLF increased according to the amount ingested. To evaluate the protective effects of bLF on the acute inflammation induced by LPS, the plasmatic concentration of the pro-inflammatory cytokine TNF-α and the gut barrier function were assessed. All the mice receiving an LPS injection presented 2 h later diarrhea. The plasmatic TNF-α concentrations at 2 h and 6 h after injection of saline or LPS at both concentrations (3 or 10 mg/kg of bw) are shown in Table 2. As no difference was observed between the two saline injected control groups ingesting LF 0.25% or LF 1% diet, only the LF1% group is presented for the control group without LPS injection in this table. Two h after LPS injection, TNF-α plasma concentration was significantly increased, and this increase was similar for the two LPS doses. At this moment (2 h), bLF ingestion did not modulate the TNF-α plasma concentration. Six h after LPS injection, the TNF-α plasma concentrations were reduced by a 4-fold factor compared to the one measured after 2 h, however, the level was still much higher than the one measured before LPS injection. After 6 h, ingestion of the diet including bLF 1% significantly reduced TNF-α plasma concentration whatever the LPS dose ingested. For the lower bLF concentration (0.25%), an inhibitory effect was observed only for the lower LPS dose (3 mg/kg of bw).

The gut barrier function was evaluated by measurement of in vivo permeability using the FD4 gavage test. Six hours after LPS injection at both doses, the plasma FD4 concentration was higher than in saline group, indicating that LPS-induced, as expected, an increase of the intestinal paracellular permeability (Table 2). Interestingly, LF 1% ingestion significantly inhibited the increased permeability by LPS at both concentration 3 and 10 mg/kg. The feces water content was significantly increased regardless of the LPS dose 2 h after injection, indicative of severe diarrhea. Ingestion of the bLF 1% diet tended to reduce the feces water content, and a significant reduction of 15% (*p* < 0.038) was observed for the mice treated with 3 mg/kg bw LPS that ingested bLF1% compared to those that did not receive bLF.

This first study showed that LPS i.p. injection at both dose (3 and 10 mg/kg bw) increased similarly plasma TNF-α, intestinal permeability and diarrhea. However, in a preliminary test, some mice injected with the higher concentration of LPS (10 mg/kg bw) died 24 h after the injection. Therefore, for the second study, we did not use the highest LPS dose. Moreover, as the diet including the highest concentration of bLF (1%) was more effective than bLF 0.25% diet to reduce LPS-induced deleterious effects, such as TNF-α plasma concentration and intestinal permeability, only the diet including 1% of bLF was used for the second study. The aim of the second study was to evaluate the protective effects of the diet including bLF 1% on intestinal alterations, 6 h and 24 h after LPS injection.

### 3.2. Protective Effect of bLF on Plasmatic TNF-α and Intestinal Permeability 6 and 24 h After LPS Injection

**Figure 2:**
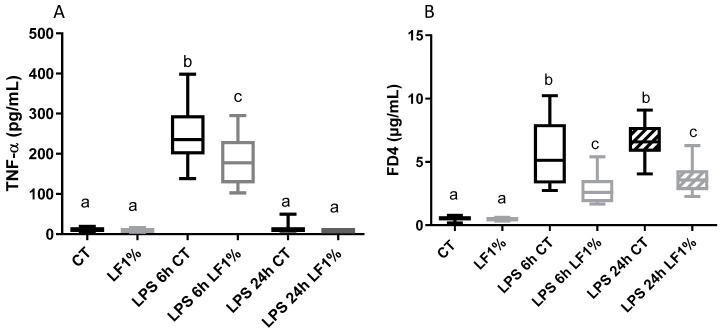
TNF-α plasmatic concentration (**A**) and in vivo intestinal permeability (**B**) in mice receiving the control diet (CT) or the bLF-supplemented diet (LF1%), 6 h or 24 h after lipopolysaccharides (LPS) injection. Data are means ± SEM (n = 10). Each group was compared with the others. Means that are significantly different (*p* < 0.05) according to the Tukey multiple comparison test have different letters.

In accordance with the first study (Table 2), Figure 2A reports that bLF ingestion reduced the marked increase in TNF-α plasma concentration 6 h after LPS injection. Twenty-four h after the LPS injection, the TNF-α plasma concentration returned to its basal level regardless of bLF ingestion. Evaluation of the in vivo intestinal permeability showed that bLF ingestion significantly inhibited the permeability increase induced by LPS, not only at 6 h after, but also at 24 h after LPS injection (Figure 2B). A correlation was observed between TNF-α plasmatic concentration and intestinal permeability (6 h: R = 0.60).

### 3.3. Preventive Effect of bLF on Ex Vivo Mucosal Permeability in Both Jejunum and Colon After LPS Injection

**Figure 3:**
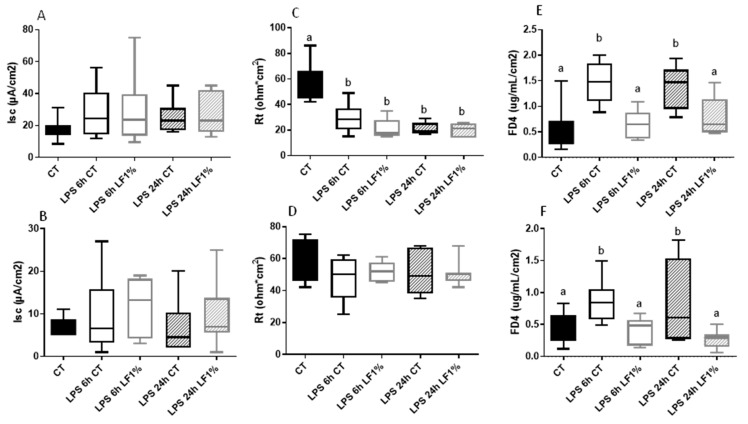
Epithelial barrier characteristics in the jejunum (**A**,**C**,**E**) and in the colon (**B**,**D**,**F**) of mice receiving the control diet (CT) or the bLF-supplemented diet (LF1%) 6 h or 24 h after lipopolysaccharides (LPS) injections. Data are means ± SEM (n = 10). Each group was compared with the others. Means that are significantly different (*p* < 0.05) according to the Tukey multiple comparison test have different letters.

To assess the potential effect of bLF supplementation on intestinal barrier function, electrophysiological and epithelial permeability measurements were performed in Ussing chambers. As we never observed any significant difference between the CT diet and the diet including LF1% for the different parameters without LPS, only the CT group is used as control before LPS injection. Evaluation of the short-circuit current (I_sc_) (Figure 3A,B) showed that LPS injection did not have any significant effect on the I_sc_ in jejunum or colon, nor bLF ingestion modified the I_sc_. The trans-epithelial resistance (R_t_, ohm*cm^2^) was reduced by LPS in the jejunum (Figure 3C) but not in the colon (Figure 3D). bLF ingestion did not have any significant effect on the trans-epithelial resistance neither in the jejunum nor in the colon. However, like what is observed in the in vivo oral test, the permeability measurement using FD4 in Ussing chambers was significantly increased in both segments when mice received LPS, although no difference was observed between 6 h and 24 h after LPS injection. These increases in permeability were significantly reduced in both intestinal segments by bLF ingestion (Figure 3E,F).

### 3.4. bLF Effects on Expression of Genes Related to Intestinal Barrier Function and Gut Homeostasis, After LPS Challenge

**Figure 4:**
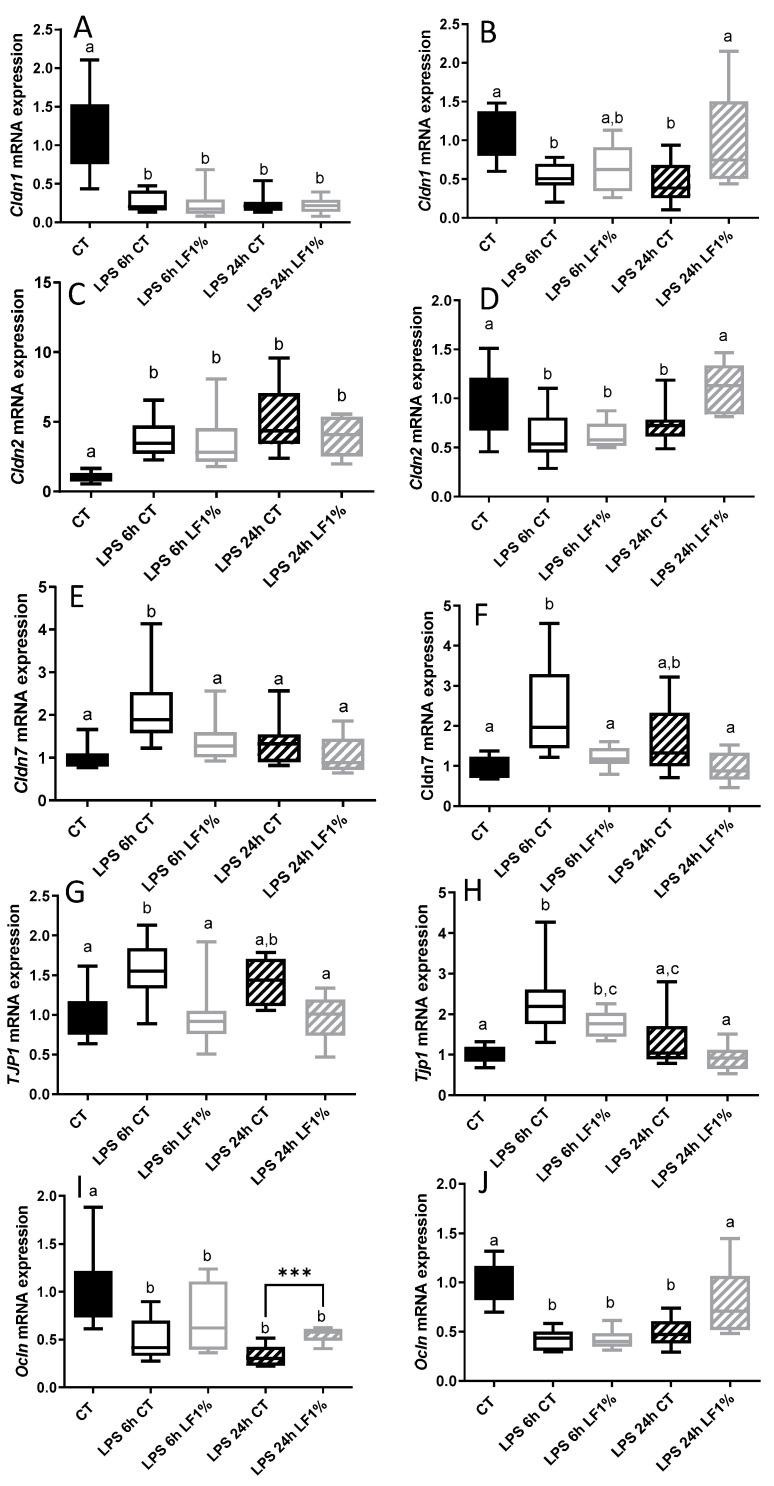
Gene expression related to intestinal barrier function in the jejunum (**A**,**C**,**E**,**G**,**I**) and in the colon (**B**,**D**,**F**,**H**,**J**) of mice receiving the control diet (CT) or the bLF-supplemented diet (LF1%) 6 h or 24 h after lipopolysaccharide (LPS) injection. Data are means ± SEM (n = 10). Means that are significantly different (*p* < 0.05) according to the Tukey multiple comparison test have different letters. A *t*-test was also used to assess the differences between two treatments at the same time point (*** *p* < 0.01).

The effects of bLF ingestion on the gene expression related to the intestinal barrier function were studied in the jejunum and the colon. In control condition, thus in the absence of LPS injection, bLF ingestion for 7 days significantly increased only *Cldn1* expression in the jejunum, values of 1.04 ± 0.13 and of 2.35 ± 0.35 (*p* = 0.0057) being reported for respectively CT and CT + LF1%. As bLF ingestion did not have any impact on all the other gene expressions studied in both segments before LPS injection, only values for the control CT group were presented in Figure 4. In the jejunum, LPS reduced *Cldn1* and *Ocln gene* expression after 6 h and 24 h, but increased expression of *Cldn2*, *Cldn7* and *Tjp1* after 6 h. However, in the colon, LPS reduced expression of *Cldn1*, *Cldn2* and *Ocln gene* after 6 h and 24 h, and increased expression of *Tjp1* and *Cldn7* after 6h. In the bLF supplemented group, 24 h after the LPS challenge, the expression of all genes returns to the control level in the colon. In the jejunum, the expression of *Cldn7* and *Tjp1* were similar to the control level, but no effect was reported for *Cldn1* and *Cldn2*, and a small increase was observed for *Ocln* 24 h after the LPS challenge in LF ingested mice. Our data indicate that LPS-induced gene expressions of tight junction protein are differently modulated by bLF ingestion in the jejunum and the colon.

**Figure 5:**
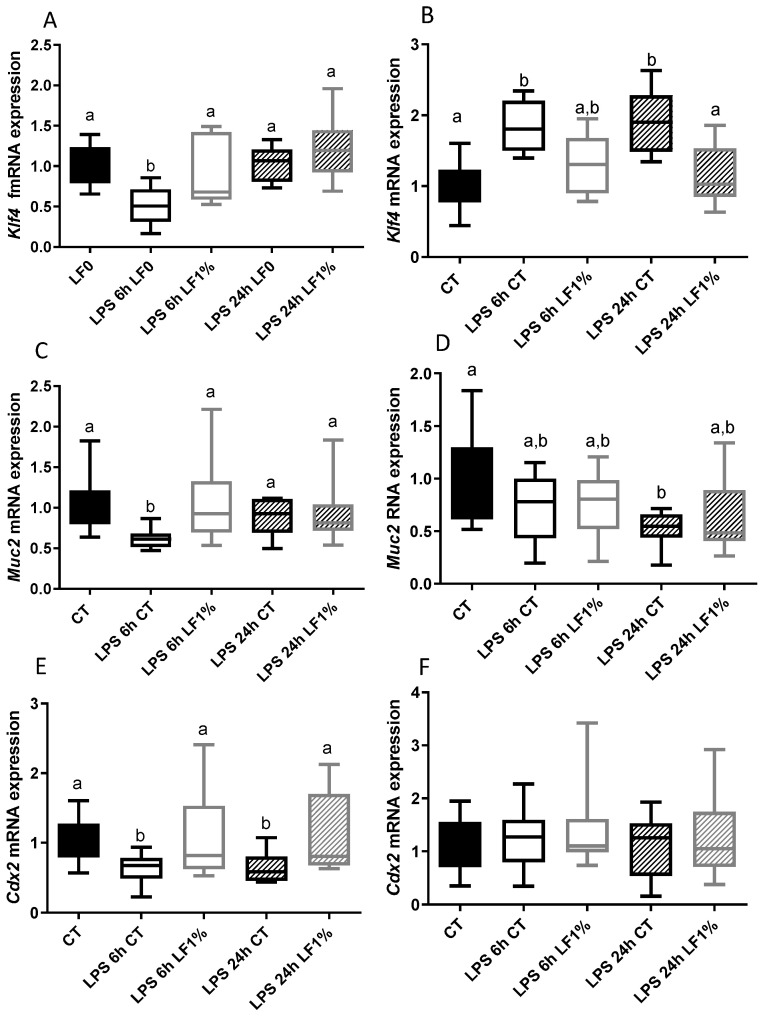
Gene expression related to gut homeostasis in the jejunum (**A**,**C**,**E**) and in the colon (**B**,**D**,**F**) of mice receiving the control diet (CT) or the bLF1%-supplemented diet (LF1%) 6 or 24 h after lipopolysaccharide (LPS) injection. Data presented are means ± SEM (n = 10). Each group was compared with the others. Means that are significantly different (*p* < 0.05) according to the Tukey multiple comparison test have different letters.

Evaluation of genes implicated in gut homeostasis is presented in Figure 5. LPS did not have the same impact on the jejunum (Figure 5A,C,E) and the colon (Figure 5B,D,F). In jejunum, LPS reduced the gene expression of *Klf4* (a marker of the goblet cells) and *Muc2* (the major intestinal mucin) 6 h after injection, as well as *Cdx2* (a gene implicated in cell differentiation) 6 h and 24 h after injection. bLF ingestion maintained these gene expressions at initial levels before LPS injection (Figure 5A,C,E). Unlike in the jejunum, in the colon, LPS increased *Klf4* expression 6 h and 24 h after LPS injection. Ingestion of bLF prevented this gene expression increase (Figure 5B). While LPS injection nor LF ingestion significantly changed *Cdx2* gene expression (Figure 5F), LPS decreased *Muc2* expression but bLF ingestion did not markedly alleviate this gene expression reduction in the colon (Figure 5D).

### 3.5. Protective Impact of bLF on Jejunal Epithelium Integrity After LPS Injection

**Figure 6:**
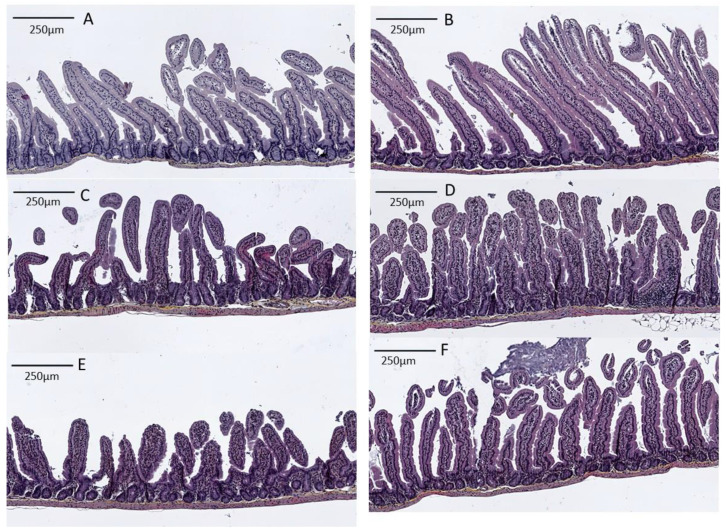
Histological examination of jejunum of control mice (**A**), of control mice receiving bLF 1% (**B**), of control mice 6 h (**C**) and 24 h (**E**) after lipopolysaccharide (LPS) injection, and of mice receiving bLF 1% 6 h (**D**) and 24 h (**F**) h after LPS injection.

As villus shortening is a parameter usually used to evaluate intestinal damages, histological examination of jejunum longitudinal sections stained with hematoxylin and eosin was performed. Figure 6 shows that bLF ingestion significantly increased villus height before LPS injection. Indeed, villus height averaged 408 ± 9 µm and 546 ± 8 µm for control and bLF groups respectively (*p* < 0.05, Figure 6A,B). Six and 24 h after LPS injection, villus shortening, clubbing and blunting were observed in the jejunum, and jejunum villus height was reduced significantly (6 h: 263 ± 7 µm, 24 h: 215± 6 µm, *p* < 0.05 (Figure 6C,E)) compared with control (408 ± 9 µm, Figure 6A). In contrast, in mice receiving bLF enriched diet, more intact villi were observed when compared with images obtained from animals not receiving bLF, and villus shortening was significantly prevented (6 h: 370 ± 7 µm, *p* < 0.05; 24 h: 334 ± 6 µm, *p* < 0.05 (Figure 6D,F)). The villus shortening was correlated strongly with the in vivo permeability (FD4) (6 h: R = 0.78, 24 h: R = 0.91), however, only a moderate correlation was reported between the villus shortening and the plasmatic TNF-α level (6 h: R = 0.48).

To better characterize the impact of bLF on jejunal epithelium, we evaluated the expression of genes related to intestinal cell growth (*Lgr5*, *Ephb2*, *Ki67)* and differentiation (*Lys5*, *Chga*, *Anpep*, *Krt20*, *SI* and *DPPIV*). bLF ingestion by the control mice did not show any significant effect on the genes selected. *Lgr5* gene expression was not modulated by LPS injection (Table 3). *Ephb2*, *Ki67*, *Lys5*, and *Krt20* gene expression were increased 6 h and 24 h after LPS injection, while *Chga* and *Anpep* gene expression were only increased 24 h after LPS injection. In addition, LPS injection decreased expression of *SI* but did not significantly modified *DPPIV* expression. In the bLF supplemented group, *Ephb2*, *Ki67*, *Lys5*, and *Krt20* gene expression were kept at the level measured before LPS administration. However, the increase in *Chga* and *Anpep* gene expression by LPS injection was not significantly modified by bLF ingestion, while *SI* expression was significantly increased by bLF ingestion after 24 h.

### 3.6. Preventive Effect of bLF on Inflammatory Gene Expression Induced by LPS in Jejunum

bLF ingestion alone did not induce any significant effect on gene expression related to inflammation. LPS injection increased gene expression of *TNF*α*, IL-1β, IL-6* and *IL-10* in the jejunum 6 h after LPS challenge (Table 4). Twenty-four h after LPS injection, *TNF*α*, IL-1β and IL-6 gene* expression were still at the same level as the one reported at 6 h, and IL-10 was reduced compared to 6 h, but remain at a higher level than the level reported before LPS Injection. bLF ingestion prevented the increase induced by LPS for *TNF*α and IL-1*β* expression 6 h after LPS injection. However, after 24 h, bLF decreased all gene expression to the control level (Table 4).

## 4. Discussion

Our data show that bLF ingestion can alleviate the systemic inflammation induced by LPS challenge and can reduce the associated intestinal damages. However, the response to LPS and the efficiency of bLF to protect the jejunum and the colon were not similar.

In accordance with previous works showing an anti-inflammatory effect of LF in endotoxemia models using LPS [17,18,19,31], our data showed that bLF ingestion decreased systemic inflammation. However, bLF ingestion was efficient for decreasing the TNF-α plasma level 6 h after LPS injection, but did not show any effect on high TNF-α plasma level observed earlier, thus 2 h after LPS injection. LF is a molecule with external surface strongly cationic which binds specifically and with high affinity to the negatively charged lipid A moiety of LPS. LF-derived cationic peptides including lactoferricins (digested product) have been shown the ability to bind LPS and a LPS neutralize activity [20]. It has been previously shown that LF and its derived peptides are able to inhibit pro-inflammatory cytokine secretion from monocytes and macrophages through different mechanisms involving sequestration of free LPS, competition with other LPS binding molecules, or inhibition of nuclear transcription factor kappa B (NF-κB) binding to TNF-α promoter, after uptake into the cells [20,32]. Moreover, a recent study showed that LF deficiency aggravates LPS-induced inflammation [33]. This suggests that endogenous LF exerts anti-inflammatory activity through modulation of chemokines and macrophage chemotaxis. Although it is unclear which mechanisms most contribute to this effect, it is possible that bLF blood concentration resulting from absorption of dietary bLF was not sufficient to suppress the large production of TNF-α induced by LPS within 2 h. Further consideration of the route, dosage, formulation and timing of LF administration is important to be considered in order to obtain a more rapid inhibitory effect than the one observed in the present study.

Modulation of intestinal permeability is a recognized marker of intestinal damage, whether the inflammation is acute or chronic. It is an important target for disease prevention and therapy, not only for intestinal diseases but also systemic diseases like infection or metabolic disorders. Indeed, self-regulating capacity of the intestinal barrier function becomes impaired in sepsis [22,23]. In the present study, dietary bLF prevented the increased intestinal permeability induced by LPS challenge, and this was correlated with a decrease in plasma TNF-α 6 h after LPS injection. Furthermore, this protective effect of LF was still observed 24 h after LPS injection. These results suggest that the increase in intestinal permeability would be partly avoided through the inhibitory effect of bLF on systemic inflammation in this model. The mucosal inflammation also compromised the epithelial barrier function. Modulation of intestinal permeability in inflammatory disease results from structural changes in tight junction proteins, which are localized on apical side of polarized epithelial cells, and which participate to the regulation of intestinal barrier function. [34,35]. The ability of bLF ingestion to preserve intestinal permeability after LPS injection can be related to *Cldn1* increased expression before LPS injection. Other markers of gut homeostasis (*Klf4*, *Muc2* and *Cdx2*) were also evaluated. The efficacy of bLF ingestion to regulate those gene expression from the LPS challenge is not only dependent on the gene considered, but our study reveals that the sensitivity of the jejunum and the colon to bLF is also different. Our data showed that the epithelial barrier characteristics and the expression of gene related to barrier function and gut homeostasis are not similarly modulated by LPS in the ileum and colon. Moreover, the response to bLF is also different which may indicate that the epithelium regeneration will not be similar in the different segment. These data are in agreement with our previous study showing that the sensitivity to bLF of the colon and ileum is not similar. In accordance with these results, our previous study showed that the responses of intestine and colon to bLF supplementation are not similar [8].

bLF ingestion was able to inhibit the intestinal inflammation induced by LPS injection, which was characterized by increased expression of genes coding for pro-inflammatory cytokines (*TNF-* α, *IL-1β* and *IL-6*) and for the anti-inflammatory cytokine *IL-10*. This latter cytokine inhibits pro-inflammatory cytokines such as TNF-α [36]. IL-10 blood levels have been shown to be correlated with inflammation severity and the development of organ failure in septic shock [37]. Twenty-four hours after LPS injection, reduced IL-10 gene expression was observed in bLF group, indicating less severe inflammation in the jejunum. The correlation between blood TNF level and intestinal permeability suggests that bLF attenuated the inflammation in the jejunum and in the colon by preventing systemic excessive LPS signaling. However, attenuation of the local inflammation by LF was indeed shown in the jejunum and in the colon in different models [15,38]. Therefore, direct anti-inflammatory action in the intestinal tract due to LF intake may contribute to the alleviation of inflammation as observed in the present study.

Histological examinations confirmed that LPS i.p. injection provoked villus shortening, clubbing and blunting, as shown in a previous study [23]. However, LF consumption prevented the villus from these damages in the jejunum. Only a moderate correlation was reported between plasmatic TNF- α level and villus shortening, supporting that bLF may have a local protective effect. Furthermore, considering that feeding for seven days with bLF increased villus height before LPS challenge, the reduction of intestinal damages by LF ingestion may involve not only the systemic inflammation alleviation but also its protective effect on intestinal tract prior to LPS injection. Indeed, our previous study showed that bLF supplementation increased jejunal villus height and reduced apoptosis, these data suggesting that bLF increases the longevity of intestinal cells [8]. Williams et al. showed that TNF- α released in the systemic circulation binds to TNF receptor-1 (TNFR1) on basolateral membrane of intestinal epithelial cells, and triggers apoptosis and shedding [23]. These results could explain the excessive cell extrusion, the villus shortening, the fluid exudation into the lumen, and may be diarrhea. Our data support the involvement of bLF against this TNF- α induced apoptosis of enterocytes to protect intestinal barrier function [28,39,40].

We previously reported that bLF supplementation stimulated growth and differentiation of mouse intestinal cells in vivo and in vitro. Moreover, in a previuos study we showed using metabolomic approaches that bLF ingestion induced higher concentrations of essential amino acids in the plasma, that allows a higher bioavailability of amino acids, in particular essential ones, to protein synthesis for epithelial regeneration. This study suggests that bLF ingestion can increase protein synthesis and play a role in the regulation of small intestine epithelial renewal. Moreover, metabolomic approaches showed that bLF ingestion induced higher concentrations of amino acids in the plasma, which could favor protein synthesis, suggesting that bLF could play, through this presumed increase in protein synthesis, a role in the regulation of small intestine epithelial renewal [7,8]. In the present study, although LPS injection induced an increased expression of genes related to intestinal cell growth (*Ephb2*) and differentiation (*Lys5* and *Krt20*) in the jejunum, these changes were not observed in mice ingesting bLF. However, *SI* expression was decreased by LPS and a small but significant increase was observed 24 h after LPS injection when mice ingested bLF. These effects of bLF on epithelial renewal represent another argument for the ability of bLF to alleviate the intestinal damage.

## 5. Conclusions

**Figure 7:**
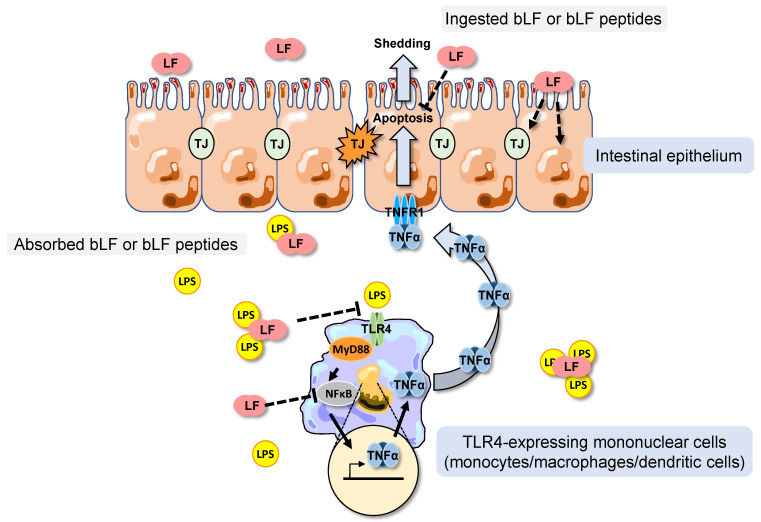
Diagram presenting the presumed pathways (dotted line) involved in the anti-inflammatory effects of bLF allowing the maintenance of the intestinal permeability. Absorbed bLF or bLF peptides can bind to LPS preventing systemic excessive LPS signaling and can inhibit the nuclear transcription factor kappa B (NF-κB). Such inhibition reduces TNF-α secretion by TLR4 (monocytes/macrophages/dendritic cells) and then consequently the interaction of TNF-α with the TNRF1 (tumor necrosis factor receptor 1). Such interaction would reduce enterocyte apoptosis and shedding, as well as tight junction (TJ) damages. Ingested bLF or bLF peptides could exert protective effects on epithelial intestinal morphology through the modulation of the expression of genes involved in epithelial cell growth and differentiation, as well as in TJ formation in the intestinal tract.

Altogether, our results show that the dietary supplementation with bLF before LPS challenge alleviates LPS-induced systemic inflammation, thereby improving the deterioration of intestinal permeability provoked by this compound. Figure 7 presents the presumed pathways involved in bLF anti-inflammatory effects, with a description of a potential mechanism by which injected LPS induces shedding in intestinal epithelial cells [23,40]. This beneficial effect of bLF on the intestinal tract may involve not only alleviation of systemic inflammation but also direct anti-inflammatory and protective effects on epithelial intestinal morphology through the modulation of the expression of genes involved in epithelial cell growth and differentiation, as well as in tight junction formation. Moreover, we also showed that the sensitivity of the jejunum and the colon to the beneficial effects of bLF against an LPS challenge was not similar.

## Figures and Tables

**Table 1:** Composition of the diets given to the mice: a control diet without LF (CT), a diet including 2.5 g/kg of bLF (LF 0.25%) and a diet including 10 g/kg of bLF (LF1%).

Ingredient (g/kg Diet)	CT	LF 0.25%	LF 1%
Bovine lactoferrin ^a^	0	2.5	10
Casein ^b^	130	127.5	120
Corn starch ^c^	630	630	630
Sucrose ^d^	103	103	103
Soybean oil ^e^	40	40	40
AIN 93M Mineral mix ^f^	35	35	35
AIN 93M Vitamins ^f^	10	10	10
Cellulose ^g^	50	50	50
Choline ^h^	2.3	2.3	2.3

^a^ Armor Protéines, Saint-Brice-en-Coglès, France. ^b^ Ingredia, Arras, France. ^c^ Cargill, Minneapolis MN, USA. ^d^ CristalCo Pro., Paris, France. ^e^ Lesieur, Asnières-sur-Seine, France. ^f^ ICN Pharmaceuticals, Orsay, France. ^g^ Prat Dumas, Couze Saint Font, France. ^h^ Jefo Nutrition, Saint-Hyacinthe, QC, Canada.

**Table 2:** Effect of different concentration of bLF and LPS on TNF-α plasma level, in vivo intestinal permeability and diarrhea.

LPS (mg/kg Bw)	LF (% of Diet)	TNF-α 2 h (pg/mL)	TNF-α 6 h (pg/mL)	Permeability 6 h FD4 (µg/mL)	Feces Water Content 2 h (%)
0	0	12.1 ± 1.4 ^a^	11.1 ± 1.5 ^a^	1.03 ± 0.09 ^a^	19.7 ± 1.4 ^a^
0	1	10.0 ± 1.0 ^a^	9.9 ± 1.1 ^a^	1.12 ± 0.12 ^a^	18.9 ± 1.4 ^a^
3	0	1361 ± 68 ^b^	363 ± 22 ^c^	8.08 ± 0.81 ^b^	72.6 ± 3.4 ^b,^
3	0.25	1281 ± 99 ^b^	235 ± 22 ^d^	9.25 ± 1.2 ^b^	72.1 ± 3.9 ^b^
3	1	1146 ± 106 ^b^	177± 12 ^e^	5.25 ± 0.41 ^c^	61.4 ± 4.1 ^b,^*
10	0	1308 ± 103 ^b^	428 ± 15 ^c^	9.50 ± 0.96 ^b^	72.2 ± 2.6 ^b^
10	0.25	1229 ± 92 ^b^	373 ± 25 ^c^	8.65 ± 1.7 ^b^	71.1 ± 3.9 ^b^
10	1	1227 ± 76 ^b^	262 ± 24 ^d^	5.62 ± 0.76 ^c^	67.2 ± 4.9 ^b^

Data are means ± SEM (n = 8). Means that are significantly different (*p* < 0.05) according to 2-way ANOVA repeated measures and the Tukey multiple comparison test have different letters. A one way ANOVA was also used to assess difference between: LPS3 LF0, LPS3 LF0.25 and LPS3 LF1% for the water content, * *p* < 0.05). bLF: bovine lactoferrin; LPS: lipopolysaccharides; TNF-α: tumor necrosis factor alpha; FD4: Fluorescein-isothiocyanate (FITC)-conjugated dextran 4000.

**Table 3:** Expression in the jejunum of genes related to the maintenance of the crypt-villous axis.

Genes	CT	LPS 6 h CT	LPS 6 h LF1%	LPS 24 h CT	LPS 24 h LF1%
*Lgr5*	1.08 ± 0.10	0.74 ± 0.10	0.83 ± 0.19	0.67 ± 0.16	0.90 ± 0.13
*Ephb2*	1.02 ± 0.07 ^a^	2.33 ± 0.30 ^b^	1.35 ± 0.09 ^a^	2.59 ± 0.25 ^b^	1.37 ± 0.11 ^a^
*Ki67*	1.02 ± 0.10 ^a^	1.86 ± 0.14 ^b^	1.09 ± 0.10 ^a^	1.74 ± 0.21 ^b,c^	1.21 ± 0.16 ^a,c^
*Lys5*	1.04 ± 0.21 ^a^	4.04 ± 0.79 ^b^	1.13 ± 0.14 ^a^	3.44 ± 0.21 ^b^	0.79 ± 0.10 ^a^
*Chga*	0.98 ± 0.07 ^a^	1.15 ± 0.08 ^a^	0.97 ± 0.09 ^a^	1.91 ± 0.24 ^b^	1.65 ± 0.10 ^b^
*Anpep*	1.00 ± 0.10 ^a^	0.98 ± 0.17 ^a^	0.82 ± 0.13 ^a^	3.34 ± 0.70 ^b^	2.44 ± 0.10 ^b^
*Krt20*	0.98 ± 0.04 ^a^	1.45 ± 0.11 ^b^	0.80 ± 0.05 ^a^	1.73 ± 0.15 ^b^	0.81 ± 0.06 ^a^
*SI*	1.00 ± 0.04 ^a^	0.28 ± 0.03 ^b^	0.37 ± 0.05 ^b,c^	0.25 ± 0.02 ^b^	0.44 ± 0.03 ^c^
*DPPIV*	1.04 ± 0.04 ^a,b^	0.79 ± 0.11 ^a^	0.88 ± 0.11 ^a,b^	1.08 ± 0.14 ^a,b^	1.34 ± 0.06 ^b^

Data are means ± SEM (n = 10). Each group was compared with the others. Means that are significantly different (*p* < 0.05) according to the Tukey multiple comparison test have different letters. CT: control diet; *Lgr5*: Leucine repeat-containing G-protein coupled receptor 5; *Ephb2:* erythropoietin-producing hepatocellular carcinoma receptor 2; *Ki67:* cellular marker of proliferation; *Lys5*: Lysine requiring; *Chga*: Chromogranin A; *Anpep:* Alanyl aminopeptidase membrane; *Krt20:* Keratin 20; *SI:* sucrase isomaltase; *DPPIV:* dipeptidyl peptidase-4; bLF: bovine lactoferrin; LPS: lipopolysaccharides; TNF-α: tumor necrosis factor alpha; FD4: Fluorescein-isothiocyanate (FITC)-conjugated dextran 4000.

**Table 4:** Expression in the jejunum of *TNF*α, *IL-1β*, *IL-6* and *IL-10* genes related to inflammation.

Genes	CT	LPS 6 h CT	LPS 6 h bLF1%	LPS 24 h CT	LPS 24 h bLF1%
*TNF*α	1.08 ± 0.16 ^a^	3.61 ± 0.31 ^b^	1.91 ± 0.20 ^a^	4.24 ± 0.41 ^b^	2.01 ± 0.15 ^a^
*IL-1β*	1.03 ± 0.10 ^a^	5.26 ± 0.73 ^b^	1.70 ± 0.24 ^a^	4.13 ± 0.76 ^b^	1.10 ± 0.16 ^a^
*IL-6*	0.99 ± 0.16 ^a^	7.00 ± 0.81 ^b^	6.03 ± 0.66 ^b^	7.00 ± 0.80 ^b^	2.62 ± 0.29 ^a^
*IL-10*	0.99 ± 0.13 ^a^	13.87 ± 2.65 ^b^	14.65 ± 2.57 ^b^	7.73 ± 1.00 ^c^	2.24 ± 0.51 ^a^

Data are means ± SEM (n = 10). Each group was compared with the others. Means that are significantly different (*p* < 0.05) according to the Tukey multiple comparison test have different letters. TNF-α: tumor necrosis factor alpha; *IL-1β:* Interleukine 1 beta; *IL-6:* Interleukine-6; *IL-10:* Interleukine-10.

## Data Availability

The raw data supporting the conclusions of this article will be made available by the authors on request.

## References

[1] Lönnerdal B. (2009). Nutritional roles of lactoferrin. Curr. Opin. Clin. Nutr. Metab. Care.

[2] Legrand D., Mazurier J. (2010). A critical review of the roles of host lactoferrin in immunity. Biometals.

[3] Kell D.B., Heyden E.L., Pretorius E. (2020). The Biology of Lactoferrin, an Iron-Binding Protein That Can Help Defend Against Viruses and Bacteria. Front. Immunol..

[4] Mayeur S., Spahis S., Pouliot Y., Levy E. (2016). Antioxid Lactoferrin, a Pleiotropic Protein in Health and Disease. Antioxid. Redox. Signal..

[5] Giansanti F., Panella G., Leboffe L., Antonini G. (2016). Lactoferrin from Milk: Nutraceutical and Pharmacological Properties. Pharmaceuticals.

[6] Legrand D. (2016). Overview of Lactoferrin as a Natural Immune Modulator. J. Pediatr..

[7] Blais A., Fan C., Voisin T., Aattouri N., Dubarry M., Blachier F., Tomé D. (2014). Effects of lactoferrin on intestinal epithelial cell growth and differentiation: An in vivo and in vitro study. BioMetals.

[8] Blais A., Lan A., Boluktas A., Grauso-Culetto M., Chaumontet C., Blachier F., Davila A.-M. (2022). Lactoferrin Supplementation during Gestation and Lactation Is Efficient for Boosting Rat Pup Development. Nutrients.

[9] Buccigrossi V., de Marco G., Bruzzese E., Ombrato L., Bracale I., Polito G., Guarino A. (2007). Lactoferrin Induces Concentration-Dependent Functional Modulation of Intestinal Proliferation and Differentiation. Pediatr. Res..

[10] Reznikov E.A., Comstock S.S., Yi C., Contractor N., Donovan S.M. (2014). Dietary bovine lactoferrin increases intestinal cell proliferation in neonatal piglets. J. Nutr..

[11] Takakura N., Wakabayashi H., Yamauchi K., Takase M. (2006). Influences of orally administered lactoferrin on IFN-γ and IL-10 production by intestinal intraepithelial lymphocytes and mesenteric lymph-node cells. Biochem. Cell Biol..

[12] Sfeir R.M., Dubarry M., Boyaka P.N., Rautureau M., Tomé D. (2004). The Mode of Oral Bovine Lactoferrin Administration Influences Mucosal and Systemic Immune Responses in Mice. J. Nutr..

[13] Togawa J.-I., Nagase H., Tanaka K., Inamori M., Nakajima A., Ueno N., Saito T., Sekihara H. (2002). Oral administration of lactoferrin reduces colitis in rats via modulation of the immune system and correction of cytokine imbalance. J. Gastroenterol. Hepatol..

[14] Li L., Ren F., Yun Z., An Y., Wang C., Yan X. (2013). Determination of the effects of lactoferrin in a preclinical mouse model of experimental colitis. Mol. Med. Rep..

[15] Hu P., Zong Q., Zhao Y., Gu H., Liu Y., Gu F., Liu H.Y., Ahmed A.A., Bao W., Cai D.J. (2022). Lactoferrin Attenuates Intestinal Barrier Dysfunction and Inflammation by Modulating the MAPK Pathway and Gut Microbes in Mice. J. Nutr..

[16] Kruzel M.L., Harari Y., Chen C.-Y., Castro G.A. (2000). Lactoferrin Protects Gut Mucosal Integrity During Endotoxemia Induced by Lipopolysaccharide in Mice. Inflammation.

[17] Kruzel M.L., Harali Y., Mailman D., Actor J.K., Zimecki M. (2002). Differential effects of prophylactic, concurrent and therapeutic lactoferrin treatment on LPS-induced inflammatory responses in mice. Clin. Exp. Immunol..

[18] Li C., Liu X., Huang Z., Zhai Y., Li H., Wu J. (2022). Lactoferrin Alleviates Lipopolysaccharide-Induced Infantile Intestinal Immune Barrier Damage by Regulating an ELAVL1-Related Signaling Pathway. Int. J. Mol. Sci..

[19] Doursout M.F., Horton H., Hoang L., Liang Y., Hwang S.A., Boyd S., Actor J.K., Kruzel M.L. (2013). Lactoferrin moderates LPS-induced hypotensive response and gut injury in rats. Int. Immunopharmacol..

[20] Puddu P., Latorre D., Valenti P., Gessani S. (2010). Immunoregulatory role of lactoferrin-lipopolysaccharide interactions. Biometals.

[21] Temiz-Resitoglu M., Kucukkavruk S.P., Guden D.S., Cecen P., Sari A.N., Tunctan B., Gorur A., Tamer-Gumus L., Buharalioglu C.K., Malik K.U. (2017). Activation of mTOR/IkappaB-alpha/NF-kappaB pathway contributes to LPS-induced hypotension and inflammation in rats. Eur. J. Pharmacol..

[22] Pool R., Gomez H., Kellum J.A. (2018). Mechanisms of Organ Dysfunction in Sepsis. Crit. Care Clin..

[23] Williams J.M., Duckworth C.A., Watson A.J., Frey M.R., Miguel J.C., Burkitt M.D., Sutton R., Hughes K.R., Hall L.J., Caamaño J.H. (2013). A mouse model of pathological small intestinal epithelial cell apoptosis and shedding induced by systemic administration of lipopolysaccharide. Dis. Model. Mech..

[24] Stephens M., von der Weid P.Y. (2020). Lipopolysaccharides modulate intestinal epithelial permeability and inflammation in a species-specific manner. Gut Microbes.

[25] Chambon-Savanovitch C., Farges M.C., Raul F., Blachier F., Davot P., Cynober L., Vasson M.P. (1999). Can a glutamate-enriched diet counteract glutamine depletion in endotoxemic rats?. J. Nutr. Biochem..

[26] Hietbrink F., Besselink M.G., Renooij W., de Smet M.B., Draisma A., van der Hoeven H., Pickkers P. (2009). Systemic inflammation increases intestinal permeability during experimental human endotoxemia. Shock.

[27] King C.J., Tytgat S., Delude R.L., Fink M.P. (1999). Ileal mucosal oxygen consumption is decreased in endotoxemic rats but is restored toward normal by treatment with aminoguanidine. Crit. Care Med..

[28] Boutry C., Matsumoto H., Bos C., Moinard C., Cynober L., Yin Y., Tomé D., Blachier F. (2012). Decreased glutamate, glutamine and citrulline concentrations in plasma and muscle in endotoxemia cannot be reversed by glutamate or glutamine supplementation: A primary intestinal defect?. Amino Acids.

[29] Beaumont M., Andriamihaja M., Armand L., Grauso M., Jaffrézic F., Laloë D., Moroldo M., Davila A.-M., Tomé D., Blachier F. (2017). Epithelial response to a high-protein diet in rat colon. BMC Genom..

[30] Vidal-Lletjós S., Andriamihaja M., Blais A., Grauso M., Lepage P., Davila A.-M., Gaudichon C., Leclerc M., Blachier F., Lan A. (2019). Mucosal healing progression after acute colitis in mice. World J. Gastroenterol..

[31] Martínez-García J.J., Canizalez-Roman A., Angulo-Zamudio U.A., Velazquez-Roman J., Flores-Villaseñor H., Valdez-Flores M.A., Rios-Burgueño E., Moran-Portela D., León-Sicairos N. (2022). Lactoferrin and Metoprolol Supplementation Increase Mouse Survival in an Experimental LPS-Induced Sepsis Model. Int. J. Pept. Res. Ther..

[32] Håversen L., Ohlsson B.G., Hahn-Zoric M., Hanson L.A., Mattsby-Baltzer I. (2002). Lactoferrin down-regulates the LPS-induced cytokine production in monocytic cells via NF-kappa B. Cell Immunol..

[33] Liu C., Peng Q., Wei L., Li Z., Zhang X., Wu Y., Wang J., Zheng X., Wen Y., Zheng R. (2023). Deficiency of Lactoferrin aggravates lipopolysaccharide-induced acute inflammation via recruitment macrophage in mice. BioMetals.

[34] Garcia-Hernandez V., Quiros M., Nusrat A. (2017). Intestinal epithelial claudins: Expression and regulation in homeostasis and inflammation. Ann. N. Y. Acad. Sci..

[35] Capaldo C.T., Powell D.N., Kalman D. (2017). Layered defense: How mucus and tight junctions seal the intestinal barrier. J. Mol. Med..

[36] Koelink P.J., Bloemendaal F.M., Li B., Westera L., Vogels E.W.M., van Roest M., Gloudemans A.K., van‘t Wout A.B., Korf H., Vermeire S. (2020). Anti-TNF therapy in IBD exerts its therapeutic effect through macrophage IL-10 signalling. Gut.

[37] Friedman G., Jankowski S., Marchant A., Goldman M., Kahn R.J., Vincent J.L. (1997). Blood interleukin 10 levels parallel the severity of septic shock. J. Crit. Care.

[38] Xie W., Song L., Wang X., Xu Y., Liu Z., Zhao D., Wang S., Fan X., Wang Z., Gao C. (2021). A bovine lactoferricin-lactoferrampin-encoding *Lactobacillus reuteri* CO21 regulates the intestinal mucosal immunity and enhances the protection of piglets against enterotoxigenic *Escherichia coli* K88 challenge. Gut Microbes.

[39] Nguyen D.N., Jiang P., Jacobsen S., Sangild P.T., Bendixen E., Chatterton D.E.W. (2015). Protective Effects of Transforming Growth Factor β2 in Intestinal Epithelial Cells by Regulation of Proteins Associated with Stress and Endotoxin Responses. PLoS ONE.

[40] Williams J.M., Duckworth C.A., Burkitt M.D., Watson A.J.M., Campbell B.J., Pritchard D.M. (2015). Epithelial Cell Shedding and Barrier Function: A Matter of Life and Death at the Small Intestinal Villus Tip. Vet. Pathol..

